# A tree-ring reconstruction of the South Asian summer monsoon index over the past millennium

**DOI:** 10.1038/srep06739

**Published:** 2014-10-23

**Authors:** Feng Shi, Jianping Li, Rob J. S. Wilson

**Affiliations:** 1 State Key Laboratory of Numerical Modeling for Atmospheric Sciences and Geophysical Fluid Dynamics, Institute of Atmospheric Physics, Chinese Academy of Sciences, Beijing 100029, China; 2 College of Global Change and Earth System Science (GCESS), Beijing Normal University, Beijing 100875, China; 3 Joint Center for Global Change Studies, Beijing 100875, China; 4 School of Geography and Geosciences, University of St Andrews, St Andrews KY16 9AL, Scotland, UK

## Abstract

The South Asian summer monsoon (SASM) is a major atmospheric synoptic climate system affecting nearly a quarter of the human population. Climate proxy data derived from tree rings, ice cores, speleothems, and other sources can all contribute to an understanding of SASM variability prior to instrumental period. Here, we develop an optimal information extraction (OIE) method, which we use to reconstruct the SASM index (SASMI) over the last millennium using 15 tree-ring chronologies. The record generated is significantly correlated (*r* = 0.7, *p* < 0.01) with the instrumental SASMI record on annual timescales; this correlation is higher than that obtained in any previous study. The reconstructed SASMI captures 18 of 26 (69%) reordered historical famine events in India over the last millennium; notably, 11 of 16 short events with durations of 1–3 years are accurately depicted in our reconstruction. Moreover, the reconstructed SASMI is positively correlated with variations in total solar irradiance (TSI) on multi-decadal timescales implying that variations in solar activity may influence the SASM. Based on the response of SASM to 34 significant volcanic events using the superposed epoch analysis, the volcanic forcing may drive a weak SASM in the second year of an eruption.

The South Asian summer monsoon (SASM; also known as the Indian summer monsoon) plays a major role in the Asian climate system, affecting nearly a quarter of the global human population. However, its pre-instrumental variability is poorly understood due to the scarcity of long-term instrumental data. Climate proxy data represent the only viable approach to exploring long-term SASM variability, and in addition have the potential to validate climate model simulations over longer time-scales.

Several high-resolution proxy records for historical drought events over the last millennium have been generated for various regions of monsoonal Asia, including those based on ice cores^1^,^2^, speleothems^3^,^4^, and tree-rings^5^,^6^,^7^. For example, high-resolution ice core data from Dasuopu on the Tibetan Plateau have demonstrated that dust and chloride concentrations reflect fluctuations in the SASM^1^. These records been used to reconstruct two well-known major drought events that occurred during recent centuries (AD 1790–1796 and AD 1876–1877). The oxygen isotopic ratios (δ^18^O) recorded in speleothems also provide excellent proxies for monsoon rainfall variability and have been widely used as indicator of monsoon intensity^3^,^4^. Recently, a dipole pattern of atmospheric precipitation is found in two speleothem δ^18^O records with near-annual resolution^8^, which provide a good representation of SASM intensity. Furthermore, a coupled climate model simulation (the Community Climate System Model version 3; CCSM 3) has provided independent confirmation that changes in the speleothem δ^18^O reflect changes in the intensity of the SASM^9^.

In SASM influenced regions, tree-ring records have often been used to reconstruct variations in precipitation^10^ and/or the Palmer Drought Severity Index (PDSI)^5^,^11^,^12^. These reconstructions have identified multi-decadal-long droughts during the 14^th^ and 15^th^ centuries. Moreover, tree-ring records from Asia have been used to reconstruct circulation indices, e.g., for the Pacific Decadal Oscillation (PDO)^13^, the Atlantic Multidecadal Oscillation (AMO)^14^, and the El Niño Southern Oscillation (ENSO)^15^,^16^,^17^. However, to our knowledge, tree-ring data have not been used to specifically reconstruct the SASMI. It is important to reconstruct the SASMI, as this index characterizes the strength of the monsoon circulation, which impacts not only regional precipitation, but is also likely to modify temperature. As tree growth is typically influenced by environmental factors such as precipitation, temperature, and soil moisture, tree-ring chronologies therefore have great potential to act as a reliable proxy for the SASMI.

Herein, we utilize a network of tree-ring chronologies, located in regions influenced by the SASM, to derive the first reconstruction of this climate index. Variations in the SASM on multiple timescales are explored using spectral analysis and Ensemble Empirical mode decomposition (EEMD). The capacity of the SASMI reconstruction to express drought events in India over the past millennium is also evaluated using historical documents. The role of natural external forcing factors, including solar activity and volcanic eruptions, in modulating the SASM are also investigated.

## Results

**Figure 1:**
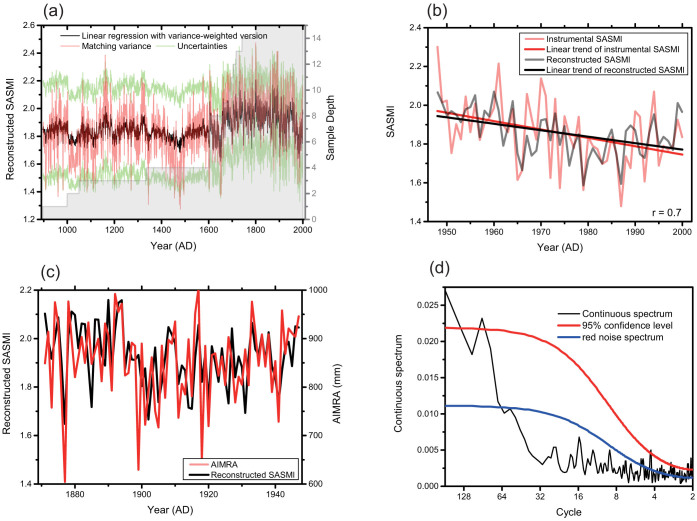
(a) The reconstructed South Asian summer monsoon index (SASMI) constructed using the matching variance method (red line) and the linear regression with variance-weighted method (black line). Green lines depicted the 2-sigma uncertainty, and grey shading indicates the sample depth. (b) Time series of the reconstructed (black line) and instrumental (red line) SASMI and associated correlation (bottom right). The straight lines are the respective linear trends. (c) Time series of the reconstructed SASMI (black line) as compared with the all-India monsoon rainfall index (AIMRI) (red line). (d) Continuous spectrum of the reconstructed SASMI (black line), its 95% confidence limits (red line), and the referenced red noise spectrum (blue line).

We synthesized 15 tree-ring chronologies from Asia to develop a temporal perspective of SASM variation over the last millennium. No significant differences were observed between composite tree-ring records generated using two alternative weighting methods: the correlation coefficient and the explained variance (Figure S1). Data assimilation methods commonly use the explained variance to establish a weighting function^18^, and therefore the variance weighted version was utilized in further analysis. Figure 1A shows two SASMI reconstructions generated using the linear regression with variance weighting and the variance matching method. The SASMI record generated using linear regression demonstrates that the weakening explanatory power of the regression equation (*r^2^*) acts to create a reduced variance back in time: for example, prior to 1605, when the sample size is less than 4 sites, *r^2^* values are only 20%. The matching variance method, on the other hand, ensures a stable variance through time. However, it does not reflect the reduced fidelity back in time. This is a typical feature of paleoclimatic reconstructions; namely, that the uncertainty increases rapidly backwards in time, as the number of proxy records becomes fewer and the signal calibration weakens. Numerous studies have examined this topic^19^; however, deciding which reconstruction method is optimal remains difficult. Therefore, in figure 1A we show results generated using both methods to reduce the bias of any single method. We utilize the variance matched version for the rest of the paper. Figure 1B illustrates the good agreement between instrumental records and our reconstructed SASMI, with a correlation coefficient of 0.70 (*n* = 53) that is significant at the 99% confidence level (*p* < 0.01). Both the instrumental and reconstructed SASMI show a statistically similar linear decrease from 1948 (p < 0.05). The all-India monsoon rainfall index (AIMRI)^20^, which is widely used to show summer monsoon activity in the Indian region, extends back to 1871, and therefore provides an opportunity to independently verify the SASMI reconstruction for the 1871–1947 period (Figure 1C). The verification results show that the squared correlation (*r^2^*) is 0.27, the reduction of error (*RE*) 0.24, and the coefficient of efficiency (*CE*) 0.24. This indicates the robustness of the reconstructed values over the independent verification period. Significant (*p* < 0.05) spectral peaks were identified at 2.0–2.2 years, 3 years, 4.3 years, and 92 years (Figure 1D), based on a continuous spectrum analysis.

Table 1 shows the correlations of the instrumental SASMI (calculated over 1948–1996) with the reconstructed SASM, the dust and chloride concentrations in the Dasuopu ice core^1^, the composite δ^18^O speleothem records from Jhumar and Wah Shikar caves^8^, and two tree-ring chronologies, one from Mae Hong Son (MHS)^12^ and one from Bidoup Nui Ba National Park (BDNP)^11^. All of these records have been published stating that they portray some aspects of the Asian monsoon. Table 2 shows the correlation between the AIMRI^20^ and these same records through the period AD 1871–1996. Only two records, our reconstructed SASMI and the composite speleothem records show significant correlations with the instrumental SASMI and the AIMRI at the 99% confidence level. The correlation of instrumental SASMI and the composite speleothem record is weaker than that of the reconstructed SASMI. Interestingly, the correlation between the reconstructed SASMI and the inverse of the composite speleothem record over the last millennium is 0.23 (*n* = 1105; *p* < 0.01), indicating that our reconstructed SASMI retains the leading modes of the SASM signal during the last millennium, similar to that expressed by the speleothem δ^18^O record. It should be noted that all other proxy records we have considered do not portray SASM variation well at the annual scale.

Most definitions place the Little Ice Age (LIA) at AD 1400–1900 and the Medieval Climate Anomaly (MCA) at AD 900–1400^21^,^22^ but there are inconsistencies in timing at regional scales. Northern hemisphere temperature reconstructions indicate that the coolest period at the hemispheric scale occurred during AD 1450–1850 and the warmest period during AD 950–1250^23^. In Asia, summer temperature reconstructions using 229 tree-ring chronologies indicate that the MCA prevailed in the period AD 850–1050 and the LIA through AD 1350–1880^24^. Annual temperature reconstructions for China based on 415 multi-proxy records pinpoint the MCA to AD 1030–1280 and the LIA to AD 1400–1700^25^, which is consistent across the different types of proxy records. It is noted that the time span of the LIA implied by the summer temperature reconstruction overlaps that inferred from the annual temperature reconstruction. The MCA (AD 1030–1280) in China was validated by other reconstructions^26^,^27^. Thus, we consider that in Asia the MCA occurred during AD 1030–1280 and the LIA during AD 1400–1700.

**Figure 2:**
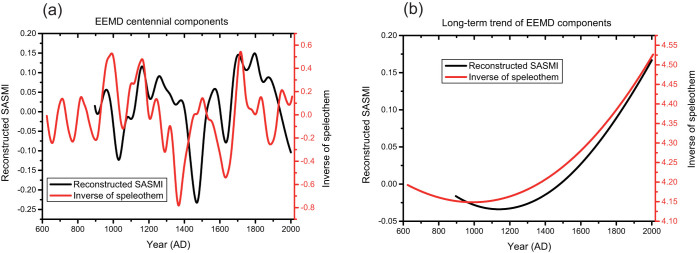
The centennial components (a) and long-term trends (b) of the reconstructed SASMI (black line) and inverse of the speleothem δ^18^O record (red line), obtained using the Ensemble Empirical mode decomposition (EEMD) method.

The EEMD method is widely used to intuitively extract the low-frequency components of variability^28^. Figure 2 illustrates the centennial modes of the reconstructed SASMI and speleothem δ^18^O records, as well as the long-term trends, extracted using the EEMD method. The centennial components show that the SASMI operated in a persistently weak phase during the LIA (Figure 2A). However, the long-term trends of both the reconstructed SASMI and speleothem record exhibit a general increase since the 13th century (Figure 2B). The suggestion in our records that a strong phase of SASM variability occurred during the MCA requires further verification, since the quality and quantity of the proxy records (1–3 tree-ring chronologies) are limited during the period and the explained variance is weak. Variations at other timescales have no obvious features (Figure S2).

The SASMI reconstruction expresses a shift in the middle of the 17^th^ century (AD 1658). Thus, in order to investigate the occurrence of extreme SASM years, we divide the record into two periods: AD 896–1658 and AD 1659–2000. For each period, we calculated the z-scores (mean of zero, standard deviation 1) of the reconstructed SASMI using the variance matching method (with stable variance). We consider SASMI values ≥ or ≤than negative/positive 1.5, respectively, to represent extreme low and high SASM years^29^,^30^. Extreme events identified by this method in the other proxies are also shown in Table S1. There are 60 extreme high and 80 low SASM years in AD 896–2000. The 30 extreme low SASM years were identified during the LIA (AD 1400–1700). The number of extreme low years (30) that occurred during the LIA is more than the number (24) occurring during the period AD 896–1399. During the LIA, 55.6% extreme low SASM events are identified in 37.4% years of the period AD 896–1700. This provides further evidence that a weak SASM existed during the LIA. Thirteen extreme high SASM years were identified during the MCA (AD 1030–1280). Table S2 highlights those events where there is agreement in all types of proxy records.

**Figure 3:**
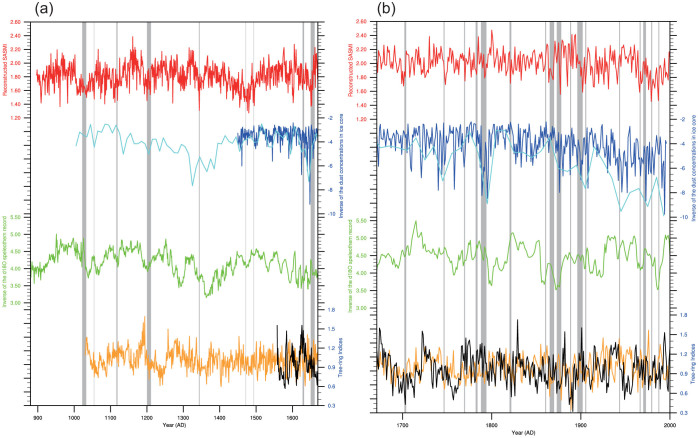
Time series of the reconstructed South Asian summer monsoon index (SASMI) (red line), the decadal (cyan line) and annual (blue line) inverse of dust concentrations in ice core record^1^, the inverse of the δ^18^O speleothem record (green line)^8^, and the tree-ring chronologies from Mae Hong Son (MHS) (black line)^12^ and Bidoup Nui Ba National Park (BDNP) (orange line)^11^ before AD 1670 (a) and after AD 1671 (b). The grey periods indicate the 26 famine events identified in India over the past millennium.

Figure 3 shows time series of the reconstructed SASMI (variance scaled), the inverse of ice core dust concentrations^1^, the inverse of the composite speleothem δ^18^O record^8^, and the two tree-ring chronologies MHS and BDNP^11^,^12^. These two chronologies (MHS and BDNP) were not used to derive the SASMI reconstruction as they expressed weak correlation with the SASMI (0.17 (1948–2005) and −0.02 (1948–2008) respectively). However, prior to the present study, these two records, which reconstruct the PDSI, were used to represent century-scale variability in the SASM^4^,^31^, based on the fact that drought in southeast Asia is generally related to the strength, timing and/or duration of the monsoon^11^,^12^. Thus, these two tree-ring chronologies are used herein as independent indicators to simply portray summer monsoon variation of a different region^4^,^31^. As a result of this prior use, and because the other proxies we consider require an independent, high-resolution record for crosschecking, we have chosen to include these records in Figure 3 despite their actual weak coherence with the SASMI.

Based on historical records in Table S2, we identified nine major famines in India before AD 1670 (Figure 3A) and 17 major famines after AD 1671 (Figure 3B). During the period AD 896–1658 the earliest famine occurs at AD 1022–1033 according to the historical documents derived from ancient India legends (Table S2), which may be subject to large dating errors^32^. In our SASMI reconstruction, there are seven extreme low SASM years in 10^th^ century, which we assign as the earliest drought events present in our record (Table S1). We infer that the earliest famine in historical documents may have occurred near AD 1017, corresponding to the 64^th^ lowest extreme year in our record, and that another famine may have occurred in AD 1032–1034 when our reconstruction contains two extreme low SASM years in AD 1032 (79^th^ lowest year) and 1034 (49^th^ lowest year). None of the other records appears to preserve a signature of the low values such as we have documented for this period. The BDNP tree-ring record (41^st^ lowest year) and the SASMI reconstruction (37^th^ lowest year) both have extreme low values in AD 1055, thus, we consider that the two records together capture a second famines, said to have occurred in Alangudi and Tanjore (in 1054) and recorded in historical documents^33^. A third famine is documented in AD 1116–1119^32^,^33^, which corresponds to an extreme low SASM year in AD 1119 (30^th^ lowest year), although again the others proxy records don't appear to indicate this event. The next historical famine, in Bombay in AD 1200, may have lasted up to 12 years^32^,^33^. In our SASMI record, there are two extreme low SASM years, AD 1200 (29^th^ lowest year) and 1209 (77^th^ lowest year), and in BDNP chronology there is an extremely low value for AD 1206 (20^th^ lowest year). A famine in AD 1343–1345^32^ appears as two lows in the SASMI during AD 1343–1344 (80^th^ and 5^th^ lowest years), a low in the speleothem record in AD 1343 (87^th^ lowest year), and one in the ice core record near AD 1335 (7^th^ lowest year). The ice core date maybe offset from the other proxies due to its coarse resolution. For the famines that are recorded in AD 1471–1472^32^ there are six corresponding extreme low years in the SASMI reconstruction during AD 1470–1476 (the 4^th^, 24^th^, 33^rd^, 46^th^, 47^th^ and 58^th^ lowest years). Famine in AD 1493–1494^32^ is recognized only in the SASMI reconstruction in AD 1492 (20^th^ lowest year) and 1495 (10^th^ lowest year). Famines recorded in Deccan and Gujarat in AD 1628–1632^32^ are identifiable in the ice core data at AD 1625 (the 3^rd^ lowest year) and in the speleothem data at AD 1630 (66^th^ lowest year). The next famine in AD 1650–1661^32^ can be found in all proxy records except for the MHS chronology. It is worth noting that eight of nine droughts associated with famines occurring before AD 1670 are successfully captured in the SASMI reconstruction, and in particular, four shorter 1–3 year events were also accurately recorded. All of these famines are associated with recognizable events in the proxy records, providing evidence that they were all caused by drought events. In the SASMI reconstruction after AD 1671 (defined as our second period; Figure 3B and Table S2), the identification of droughts associated with famine events is similarly successful, with 10 of 17 events recorded. In particular, seven 1–3 year events were accurately captured in our reconstruction. It is very difficult to accurately identify these shorter events in the ice core and speleothem records because of their dating uncertainties.

**Figure 4:**
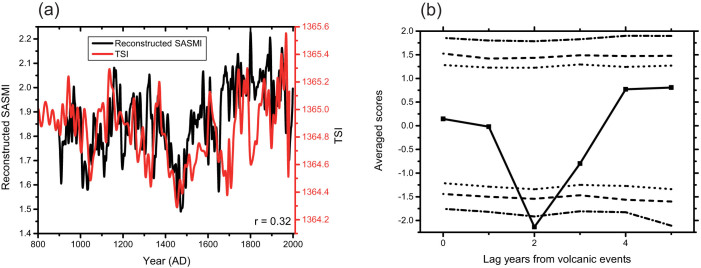
(a) Time series of the reconstructed South Asian summer monsoon index (SASMI) and total solar irradiance (TSI) over the last millennium. (b) Superposed Epoch Analysis results applied to the SASMI reconstruction response to 34 significant volcanic events with 90%, 95% and 99% confidence limits of the mean given as dashed, dotted, and dashed-dotted lines, respectively.

The previous section has shown that our SASMI reconstruction shows reasonable fidelity with the historical famine record. However, an understanding of the forcing mechanisms that drive dynamic phenomena such as the SASM is a critical step in palaeoclimatology. External forcings, such as solar activity, volcanic events and greenhouse gases, are possible factors that may influence the SASM. Herein, we undertake a preliminary investigation exploring whether external forcings might have influenced the SASM during the pre-Industrial Period. Reconstructions of total solar irradiance (TSI), which representing the intensity of solar activity, differ somewhat in their resolution and magnitude depending on whether they are physically based or extrapolated from physically based data^34^. However, most of the reconstructions depict similar trends in solar activity over the last millennium. We compared our reconstructed SASMI with TSI reconstructed on the basis of Antarctic ^10^Be^35^ (Figure 4A; both indices smoothed using a 10-year loess filter), and found a positive correlation over the period AD 896–1982 (*r* = 0.32, the effective degrees of freedom = 28) at the 95% confidence level. Figure 4B shows the SASM response to 34 significant Northern Hemisphere volcanic events during AD 1111–1976^36^ using a superposed epoch analysis (SEA). The SEA results indicate that a negative response, statistically significant at the 99% confidence level, occurred within the second year after the eruptions.

## Discussion

During a drought event, low precipitation causes low soil moisture and high temperatures leads increased evaporation, resulting in increased water stress in trees and reduced rates of cell division, causing the formation of narrow rings^37^. Other factors, e.g. the fire^38^ and earthquakes^39^ can also result in narrow rings. However, by selecting appropriate samples and constructing a composite record using multiple tree-ring chronologies we can reduce or avoid the influence of the above factors. Famines, on the other hand, are not solely caused by drought, but maybe induced by human factors such as war or tyranny. The SASMI accurately captures 18 of 26 famine events recorded in historical documents; 11 of 16 shorter 1–3 year events are especially well depicted in our reconstruction. Note that not all low values in the SASMI imply famine, because a weak monsoon does not necessarily result in famine. Moreover, we cannot exclude the possibility that some extreme low SASM years did coincide with famine, but that these famines are not recorded in historical documents. For example, the reconstructed SASMI indicates that five severe drought years occurred in the middle of the fifteenth century (AD 1453, 1455, 1456, 1457, and 1459) and may have caused a consecutive decadal drought event, which is verified by the ice record, but not recorded in historical documents. Thus, the SASMI reconstruction provides a reference that maybe used to identify periods of incomplete information in historical documents.

Based on our power spectrum analysis, we suggest that the characteristics of the SASMI on multiple timescales maybe related to various forms of internal forcing. The 2–3 year periodicities, which are often observed in Chinese tree-ring reconstructions^40^,^41^, may be related to the tropospheric biennial oscillation (TBO). Quasi 4-years cycles are likely to correspond to ENSO variability, as El Niño events arising through east–west displacement of the ascending and descending branches of the Walker circulation affect the SASM^42^. Severe droughts always coincide with El Niño events^43^. Moreover, a robust average for ENSO periodicity is ca. 4 years, as El Niño and La Niña episodes often appear every 3–5 years^44^. The multi-decadal (92-year) cycle is apparent in the reconstructed SASM variability, and is also is found in the Dandak δ^18^O speleothem record^45^. The 92-year cycle may be the major cycle of multi-decadal SASM variability, corresponding to the Gleissberg frequency band of solar activity, which is strongly manifested in multiple indicators^46^. The multi-decadal variability in the SASM over the last millennium may be driven by changes in solar irradiance, which affects the thermal contrast between the land and ocean in the SASM region.

In terms of external forcing factors driving variability in the SASM, we have demonstrated that both solar force and volcanic activity have statistically significant relationships with the reconstructed SASMI. We suggest that the relationship between SASM and solar activity is related to variations in solar radiation, which cause an increase in the north–south (land–sea) temperature gradient in areas affected by the SASM, with northern land regions warming more rapidly than southern ocean regions. These variations, in turn, induce stronger SASM winds. Thus, solar activity may be a critical driving force of SASM intensity at multidecadal time scales, through variations in the land–ocean thermal contrast. There is a statistically significant negative response in the SASMI to volcanic forcing in the second year following a volcanic event at the 99% confidence level. This provides evidence for the aerosols derived from volcanic eruptions might lead to drought in South Asia monsoon region, consistent with the observation^47^, the model simulation^48^,^49^,^50^ and the proxy reconstruction^51^,^52^. A possible mechanism is that the volcanic aerosol cooling effect causes the larger cooling over land than the surrounding oceans due to their different heat capacity, and has a decline of the latent heat flux over ocean and the sensible heat flux over land in Asian monsoon region^49^,^50^. All imply that a reduced land-sea thermal contrast in Asian monsoon region induces a weak SASM.

## Methods

### Data

Four common instrumental precipitation datasets were used in this study: the NOAA Precipitation Reconstruction over Land (NOAA PREC/L)^53^, the Global Precipitation Climatology Centre Reanalysis version 6 (GPCC Reanalysis V6)^54^, the Climate Prediction Center Merged Analysis of Precipitation (CMAP)^55^, and the Global Precipitation Climatology Project version 2.2 combined precipitation dataset (GPCP V2.2)^56^. The common period of the first two datasets (NOAA PREC/L and GPCC Reanalysis V6) is 1948–2010, and that of the latter two datasets (CMAP and GPCP V2.2) is 1979–2010.

We used the SASMI determined during the 1948–2010 period as a calibration dataset. SASMI is defined as the seasonal (June–July–August–September; JJAS) area-averaged dynamical normalized index at 850 hPa in the South Asian domain (5°–22.5°N, 35°–97.5°E)^57^. This index is available on the website of the second author (http://ljp.lasg.ac.cn/dct/page/65576).

The all-India monsoon rainfall index (AIMRI), which is widely used to show summer monsoon activity in the Indian region, extends back to 1871, and therefore provides a longer reference timescale than the SASMI. The AIMRI, which is independent of the SASMI, is based on a homogeneous rainfall dataset of 306 rain gauges in India, developed by the Indian Institute of Tropical Meteorology^20^. The correlation coefficient between the instrumental SASMI and AIMRI over the period 1948–2000 is 0.62, significant at the 99% level (*p* < 0.01, n = 53; Figure S3). The AIMRI therefore provides a complementary dataset for verification of the reconstructed SASMI prior to 1948.

18 tree-ring chronologies were selected according to their significant correlations with the SASMI (*p* < 0.1). Correlations between the SASMI and the four instrumental precipitation datasets were then calculated (Figure S4). Only those tree-ring chronologies that were located in spatial grids where the correlation coefficients exceeded the 90% confidence level were finally used for the analysis. Using this screening process, 15 tree-ring chronologies were selected to reconstruct the SASMI. Figure S4 shows the positive or negative correlations of these 15 chronologies with the SASMI.

Of the 15 tree-ring chronologies selected for the analysis, eight are available in the supplement of the Past Global ChangES (PAGES) 2 k Consortium paper^23^, one is accessible from the International Tree-Ring Data Bank (ITRDB), maintained by the NOAA World Data Center for Paleoclimatology, two are archived in a book^58^, one is shared by Prof. Yang^59^, one is obtained from Dr. Sano^16^, and two are provided by Dr. Xu^15^,^17^. These tree-ring chronologies that are significantly correlated with SASM variability (*p* < 0.1), are a scarce and precious resource. The details of each chronology are listed in Table S3.

The history of famines in South Asia has been recorded in historical documents. Over the last millennium, 24 notable famines have occurred in India (see Table S2). Generally, a reduction in food production over successive years was the immediate cause of the famines, although drought and war are both common causes of reduced production^60^. However, if extreme environmental conditions at the time of a famine are recorded in a natural proxy record, we have reason to believe that the famine was caused by a drought event, especially since an important criterion for the development of the natural proxy records is the absence of interference by human activities.

### Analytical methods

The composite plus scale (CPS) framework is convenient and widely used in paleoclimate reconstruction^19^. In this process, proxy records are weighted and combined before calibration. A linear transfer function is then established based on the composite record, with the noise in the proxy record obeying that of a normal distribution. Thus, combining multiple records will minimize noise and maximize the common climate signal. The common signal from multiple proxy records may decrease the uncertainty contributed by a specific proxy record.

The OIE method was proposed to reconstruct the SASMI, and to reasonably take into account the signal-to-noise ratio of every record by optimizing the weights of the records. Term *p_i_*_,*t*_ of the proxy record denotes the *i^th^* proxy record at year *t*. The composite record at year *t* is called *P_t_*. The correlation coefficient between the *i^th^* proxy record and its allocated instrumental record during the entire available period is represented by *r_i_*. The weight of every record can be given by two methods, as follows. Method 1 uses the correlation coefficient (Eq. 1) to estimate the weight: 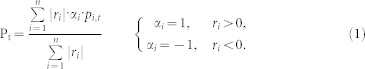
Method 2 uses the explained variance (Eq. 2) to estimate the weight: 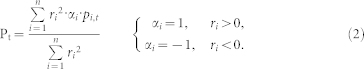
The parameter *α* is a constant, and the explained variance is calculated using the square of the correlation coefficient.

The next step is to rebuild the transfer functions using two approaches, linear regression (Eq. 3) and variance matching (Eq. 4). We assume that *I_t_* is represented by the reconstructed SASMI in the year *t* by the linear regression: 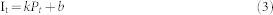
The matching variance is given by: 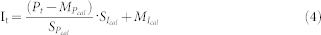
where *k* is an equation coefficient, *b* is the intercept of the model, *M_Pcal_* and *M_Ical_* are the means of the combined proxy record and the instrumental data during the calibration period, respectively, and *S_Pcal_* and *S_Ical_* are the standard deviations of the combined proxy record and the instrumental data during the calibration period, respectively.

The AIMRI data in AD 1871–1947 was used to validate the reconstructed SASMI. The verification skills were assessed through the square of the Pearson product-moment correlation coefficient (*r^2^*), the reduction of error (*RE*), and the coefficient of efficiency (*CE*). The uncertainty (*U*) was estimated using the standard deviation (*std*) of the instrumental SASMI and the correlation coefficient (*r*) between the reconstructed and instrumental SASMI using the equation: 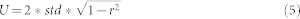
The criteria defining whether the events in the reconstructed SASMI record were associated with historical records of famines are as follows. Since the tree-ring may take a year to respond to a drought event, a narrow ring may occur in the second year after a drought event. It may also be true that a famine will occur in the second year after a drought event. Thus, if there is 1 year offset between a drought event and an historical famine, we consider that our reconstruction has captured the event. Moreover, if a famine event persists several years to decades within which there is one extreme low SASM year at least we also consider that our reconstruction has captured the event.

The relationship between two variables in this study is all examined using the Pearson's linear correlation coefficient. The p-value for Pearson's correlation is computed using a Student's t distribution, and the confidence level with the two-tailed test.

## Author Contributions

J.L. and F.S. designed the research, J.L., F.S. and R.J.S.W. contributed to interpreting the results and writing the main text. F.S. collected the tree-ring chronologies and plotted the figures. All authors reviewed the manuscript.

## Additional information

**Data archive:** The SASMI reconstruction is archived at the World Data Center for Paleoclimatology.

## Supplementary Material

Supplementary Information Supplementary Information

Supplementary Information Dataset 1

## Figures and Tables

**Table 1:** Correlation coefficients between the instrumental South Asian summer monsoon index (SASMI) and the reconstructed SASMI, the dust and chloride concentration ice core record, the δ^18^O speleothem record, and two tree-ring chronologies, for the period AD 1948–1996 (*n* = 49)

	Instrumental SASMI	Reconstructed SASMI	Dust concentrations in ice core	Chloride concentrations in ice core	δ^18^O record in Speleothem	MHS	BDNP
Instrumental SASMI	1.00	0.71**	0.21	−0.02	−0.38**	0.18	−0.04
Reconstructed SASMI		1.00	−0.07	−0.02	−0.48**	−0.03	0.00
Dust Concentrations in ice core			1.00	0.56**	−0.11	0.21	−0.10
Chloride Concentrations in ice core				1.00	0.14	0.16	0.07
δ^18^O record in Speleothem					1.00	−0.20	−0.07
MHS						1.00	−0.38**
BDNP							1.00

**Indicates correlation coefficients statistically significant at the 99% confidence level.

**Table 2:** Correlations between the all-India monsoon rainfall index (AIMRI) and the reconstructed South Asian summer monsoon index (SASMI), the dust and chloride concentration ice core records, the δ^18^O speleothem records, and two tree-ring chronologies, for the period AD 1871–1996 (*n* = 126)

	AIMRI	Reconstructed SASMI	Dust concentrations in ice cores	Chloride concentrations in ice cores	δ^18^O speleothem record	MHS	BDNP
AIMRI	1.00	0.53**	−0.06	−0.03	−0.23**	0.08	−0.11
Reconstructed SASMI		1.00	−0.12	−0.10	−0.18*	−0.02	0.04
Dust concentrations in ice core			1.00	0.59**	−0.19*	0.15*	0.03
Chloride concentrations in ice core				1.00	−0.08	0.22*	0.06
δ^18^O record in Speleothem					1.00	−0.24**	0.02
MHS						1.00	−0.13
BDNP							1.00

**Indicates correlation coefficients statistically significant at the 99% confidence level;

*Indicates correlation coefficients statistically significant at the 90% confidence level.
