# Supplementary material for: A tree-ring reconstruction of the South Asian summer monsoon index over the past millennium
Source: Sci Rep. 2014 Oct 23;4:6739. doi: 10.1038/srep06739 (PMC4206867; doi:10.1038/srep06739)
Supplement: Supplementary Information [file srep06739-s1.docx]

**Supplementary Information for**

**‘A tree-ring reconstruction of the South Asian**

**summer monsoon index over the past millennium’**

Feng Shi^1^, Jianping Li^2, 3, *^, Rob J. S. Wilson^4^

*^1^State Key Laboratory of Numerical Modeling for Atmospheric Sciences and Geophysical Fluid Dynamics, Institute of Atmospheric Physics, Chinese Academy of Sciences, Beijing 100029, China, ^2^College of Global Change and Earth System Science (GCESS), Beijing Normal University, Beijing 100875, China,^3^Joint Center for Global Change Studies, Beijing 100875, China, ^4^School of Geography and Geosciences, University of St Andrews, St Andrews KY16 9AL, Scotland, UK*

*To whom correspondence should be addressed.

E-mail: ljp@bnu.edu.cn

This file includes three Supplementary Tables and four Supplementary Figures. Table S1 shows the ranking information of low/high extreme values of the reconstructed South Asian summer monsoon index (SASMI), and the low extreme values of the other proxy records.Table S2 lists 26 famines in India during the last millennium.Table S3provides the information of 15 tree-ring chronologies. Figure S1 shows the reconstructed SASMI during the last millennium using two methods to estimate the weight of record. Figure S2 shows the Ensemble Empirical Model Decomposition (EEMD) analysis of the reconstructed SASMI and the inverse of speleothem δ^18^O record. Figure S3 shows comparison of the instrumental SASMI and all-India monsoon rainfall index. Figure S4 illustrates the screened tree-ring chronologies at the girds, where the instrumental precipitations are significantly related to the SASMI.

Table S1. The ranking information of low/high extreme values of the time series of the reconstructed SASMI, and the low extreme values of the inverse of the ice core dust record^1^, the inverse of the oxygen isotopic ratios (δ^18^O) ofthe composite speleothem record^2^, and the two tree-ring chronologies (MHS and BDNP)^3, 4^.

| Rank | High  SASMI | Low SASMI | Annual  Ice-core | Decadal  Ice-core | Speleothem | BDNP | MHS |
| --- | --- | --- | --- | --- | --- | --- | --- |
| 1 | 1161 | 1979 | 1648 | 1325 | 1363 | 1889 | 1580 |
| 2 | 1800 | 1987 | 1994 | 1645 | 1364 | 1878 | 1579 |
| 3 | 1605 | 1480 | 1625 | 1993 | 1365 | 1404 | 1611 |
| 4 | 1167 | 1476 | 1641 | 1365 | 1366 | 1865 | 1577 |
| 5 | 1577 | 1344 | 1973 | 1945 | 1370 | 1635 | 1589 |
| 6 | 1316 | 1455 | 1795 | 1385 | 1367 | 1236 | 1891 |
| 7 | 1890 | 1877 | 1461 | 1335 | 1374 | 1243 | 1887 |
| 8 | 1894 | 961 | 1789 | 1975 | 1369 | 1403 | 1703 |
| 9 | 1066 | 1651 | 1952 | 1795 | 1359 | 1364 | 1757 |
| 10 | 1893 | 1495 | 1876 | 1355 | 1368 | 1610 | 1949 |
| 11 | 1576 | 1653 | 1946 |  | 1380 | 1425 | 1889 |
| 12 | 1610 | 1966 | 1740 |  | 1362 | 1913 | 1713 |
| 13 | 1155 | 1902 | 1758 |  | 1360 | 1964 | 1888 |
| 14 | 953 | 1084 | 1908 |  | 1379 | 1504 | 1822 |
| 15 | 1163 | 1481 | 1991 |  | 1371 | 1526 | 1826 |
| 16 | 1169 | 982 | 1545 |  | 1361 | 1647 | 1689 |
| 17 | 1730 | 1271 | 1640 |  | 1372 | 1615 | 1756 |
| 18 | 1325 | 909 | 1590 |  | 1872 | 1747 | 1956 |
| 19 | 1785 | 1602 | 1657 |  | 1986 | 1634 | 1955 |
| 20 | 1184 | 1492 | 1649 |  | 1873 | 1206 | 1851 |
| 21 | 1321 | 1976 | 1634 |  | 1987 | 1585 | 1873 |
| 22 | 1328 | 1112 | 1790 |  | 1396 | 1651 | 1696 |
| 23 | 1801 | 1931 | 1942 |  | 1876 | 1582 | 1760 |
| 24 | 1241 | 1474 | 1900 |  | 1373 | 1969 | 1699 |
| 25 | 1158 | 1468 | 1721 |  | 1875 | 1919 |  |
| 26 | 967 | 1456 | 1961 |  | 1643 | 1239 |  |
| 27 | 1575 | 1983 | 1880 |  | 1800 | 1405 |  |
| 28 | 1157 | 1663 | 1796 |  | 1642 | 1402 |  |
| 29 | 936 | 1200 | 1967 |  | 1985 | 1066 |  |
| 30 | 1879 | 1119 | 1947 |  | 1624 | 1583 |  |
| 31 | 1606 | 1436 | 1982 |  | 1874 | 1636 |  |
| 32 | 1535 | 1915 | 1888 |  | 1376 | 1581 |  |
| 33 | 1282 | 1473 | 1744 |  | 1377 | 1804 |  |
| 34 | 1871 | 1914 | 1976 |  | 1358 | 1692 |  |
| 35 | 1002 | 1459 | 1979 |  | 1615 | 1417 |  |
| 36 | 1283 | 1484 | 1567 |  | 1288 | 1597 |  |
| 37 | 1880 | 1055 | 1930 |  | 1859 | 1232 |  |
| 38 | 1658 | 1980 | 1710 |  | 1799 | 1897 |  |
| 39 | 971 | 1885 | 1792 |  | 1616 | 1310 |  |
| 40 | 1267 | 1419 | 1978 |  | 1375 | 1089 |  |
| 41 | 1367 | 1701 |  |  | 1797 | 1055 |  |
| 42 | 1252 | 1073 |  |  | 1871 | 1771 |  |
| 43 | 1875 | 1108 |  |  | 1293 | 1777 |  |
| 44 | 1164 | 1438 |  |  | 1295 | 1621 |  |
| 45 | 1186 | 1992 |  |  | 1625 | 1368 |  |
| 46 | 1166 | 1470 |  |  | 1401 | 1764 |  |
| 47 | 1366 | 1471 |  |  | 1403 | 1978 |  |
| 48 | 1735 | 1748 |  |  | 1856 | 1235 |  |
| 49 | 1140 | 1034 |  |  | 1667 | 1503 |  |
| 50 | 1886 | 1110 |  |  | 1294 | 1347 |  |
| 51 | 1887 | 1926 |  |  | 1858 | 1825 |  |
| 52 | 1862 | 1466 |  |  | 1287 | 1409 |  |
| 53 | 1521 | 1941 |  |  | 1292 | 1240 |  |
| 54 | 1574 | 1982 |  |  | 1857 | 1088 |  |
| 55 | 1125 | 1603 |  |  | 1378 | 1480 |  |
| 56 | 1223 | 1905 |  |  | 1402 | 1374 |  |
| 57 | 1960 | 1699 |  |  | 1860 | 1565 |  |
| 58 | 1320 | 1472 |  |  | 1668 | 1843 |  |
| 59 | 1171 | 1968 |  |  | 1798 |  |  |
| 60 | 1948 | 1920 |  |  | 1614 |  |  |
| 61 |  | 910 |  |  | 1648 |  |  |
| 62 |  | 1821 |  |  | 1662 |  |  |
| 63 |  | 1986 |  |  | 1397 |  |  |
| 64 |  | 1017 |  |  | 1611 |  |  |
| 65 |  | 1453 |  |  | 1297 |  |  |
| 66 |  | 1991 |  |  | 1630 |  |  |
| 67 |  | 1864 |  |  | 1381 |  |  |
| 68 |  | 908 |  |  | 1612 |  |  |
| 69 |  | 1008 |  |  | 1593 |  |  |
| 70 |  | 1600 |  |  | 1663 |  |  |
| 71 |  | 1965 |  |  | 1594 |  |  |
| 72 |  | 931 |  |  | 1647 |  |  |
| 73 |  | 1086 |  |  | 1988 |  |  |
| 74 |  | 1482 |  |  | 1296 |  |  |
| 75 |  | 1457 |  |  | 1299 |  |  |
| 76 |  | 920 |  |  | 1870 |  |  |
| 77 |  | 1209 |  |  | 1290 |  |  |
| 78 |  | 1444 |  |  | 1595 |  |  |
| 79 |  | 1032 |  |  | 1669 |  |  |
| 80 |  | 1343 |  |  | 1877 |  |  |
| 81 |  |  |  |  | 1291 |  |  |
| 82 |  |  |  |  | 1592 |  |  |
| 83 |  |  |  |  | 1384 |  |  |
| 84 |  |  |  |  | 1286 |  |  |
| 85 |  |  |  |  | 1354 |  |  |
| 86 |  |  |  |  | 1626 |  |  |
| 87 |  |  |  |  | 1343 |  |  |
| 88 |  |  |  |  | 1400 |  |  |
| 89 |  |  |  |  | 1356 |  |  |
| 90 |  |  |  |  | 1646 |  |  |
| 91 |  |  |  |  | 1670 |  |  |
| 92 |  |  |  |  | 1661 |  |  |
| 93 |  |  |  |  | 1923 |  |  |
| 94 |  |  |  |  | 1610 |  |  |

Table S2. List of the 26 famines in India during the last millennium, ordered by year, where there is agreement in all types of proxyrecords.

| **Date** | **Drought events/**  **Locality** | **Source** | **SASMI** | **Ice-**  **core** | **Speleo-**  **them** | **Tree-ring** | | |
| --- | --- | --- | --- | --- | --- | --- | --- | --- |
|  |  |  |  |  |  | BNDP | MHS | |
| **1022-1033** | Great famine in India | ^5, 6^ | * |  |  |  |  | |
| **1054** | Famines in Alangudi and Tanjore | ^7, 8^ | * |  |  | * |  | |
| **1116-1119** | Famine in Deccan | ^6, 7^ | * |  |  |  |  | |
| **1200-1211** | Famines in Tiruppamburam and Tanjore | ^6, 7^ | * |  |  | * |  | |
| **1343-1345** | Great famine | ^6, 9^ | * | * | * |  |  | |
| **1471-1472** | Bijapur famine | ^6^ | * |  |  |  |  | |
| **1493-1494** | Bombay famine | ^6, 7^ | * |  |  |  |  | |
| **1628-1632** | Famines in Deccan and Gujarat | ^5, 6, 10^ |  | * | * |  |  | |
| **1650-1661** | Famines in Ahmedabad, Bengal and Bombay | ^6, 11^ | * | * | * | * |  | |
| **1702-1704** | Famine in Deccan | ^12, 13^ | * | * |  |  | * | |
| **1746-1747** | Famine in Bombay | ^6^ | * | * |  | * |  | |
| **1769-1770** | Bengal famine | ^5, 6, 14, 15, 16^ |  |  |  | * |  | |
| **1782-1784** | Madras city and surrounding areas, Chalisa famine | ^6, 17^ |  |  |  |  |  | |
| **1788-1794** | The Doji Bara or Skull famine, East India Drought | ^17^ |  | * | * |  |  | |
| **1820-1822** | Upper Sind famine | ^6, 18^ | * |  |  |  | * | |
| **1860-1861** | Upper Doab famine | ^19, 20^ |  |  | * |  |  | |
| **1865-1870** | Famines in Orissa and Rajputana | ^20, 21^ | * |  | * | * |  | |
| **1873-1878** | Southern India famine | ^5, 14, 20, 22, 23^ | * | * | * | * | | * |
| **1888-1889** | Late Victorian droughts | ^22^ |  | * |  | * | | * |
| **1896-1902** | Famine in India | ^15, 20, 23, 24^ | * | * |  | * | |  |
| **1905-1906** | Famines in Bombay | ^21^ | * | * |  |  | |  |
| **1943-1944** | Bengal Famine | ^5, 21^ |  | * |  |  | |  |
| **1966-1967** | Bihar famine | ^8, 21, 25^ | * | * |  |  | |  |
| **1970-1973** | Maharashtra drought | ^5, 25^ |  | * |  | * | |  |
| **1979-1980** | West Bengal drought | ^26^ | * | * |  | * | |  |
| **1987** | Gujarat Famine | ^27^ | * | * | * |  | |  |

*Indicates that the famine corresponds to an extremely low local value in the natural proxy records (otherwise, no symbol is given).

Table S3. List of 15 tree-ring chronologies used to derive the SASMI reconstruction, showing their geographic locations, the period covered by each record, and their sources.

| No. | Site | Longitude | Latitude | Start year | End year | Author/Source |
| --- | --- | --- | --- | --- | --- | --- |
| 1 | AT-UKHLWD | 85.22 | 50.09 | 1605 | 1994 | ^28^ |
| 2 | AT-UKHMXD | 85.22 | 50.09 | 1605 | 1994 | ^28^ |
| 3 | CH-DLH1 | 97.14 | 37.28 | 1050 | 2001 | ^28^ |
| 4 | CH-DULAJP | 98.05 | 36.18 | 896 | 1993 | ^28^ |
| 5 | JP-KUNASH | 145.36 | 43.53 | 1603 | 2000 | ^28^ |
| 6 | KZ-ATBEST | 76.21 | 41.13 | 1742 | 2005 | ^28^ |
| 7 | MG-URGNPS | 110.33 | 48.35 | 1664 | 1996 | ^28^ |
| 8 | PK-MOR4JU | 75.05 | 36.35 | 1336 | 1990 | ^28^ |
| 9 | VM-MCCHFH2 | 104.06 | 21.4 | 1705 | 2003 | ^29^ |
| 10 | Wat Chan (th006) | 98.14 | 19.01 | 1695 | 2004 | ITRDB* |
| 11 | Liah172 | 87.09 | 48.3 | 1717 | 1981 | ^30^ |
| 12 | Liah173 | 87.45 | 48.22 | 1685 | 1981 | ^30^ |
| 13 | Qamdo | 96.57 | 31.1 | 1000 | 2010 | ^31^ |
| 14 | LS-MCCPL | 103.55 | 20.17 | 1801 | 2001 | ^32^ |
| 15 | PhuLeuy mountain | 103.55 | 20.17 | 1605 | 2002 | ^33^ |

* ITRDB means International Tree-Ring Data Bank in National Climatic Data Center (NCDC).

Figure S1. The reconstructed South Asian summer monsoon index (SASMI) during the last millennium using two methods to estimate the weights of record, the first based on the correlation coefficient (black line) and the second on the explained variance (red line); the uncertainties are given in green; the gray area indicates the explained variance of the reconstructed SASMI.

Figure S2. Ensemble Empirical Model Decomposition (EEMD) analysis of the reconstructed South Asian summer monsoon index (SASMI) and the inverse of speleothem record at the interannual (A), interdecadal (B) and multidecadal (C) components.


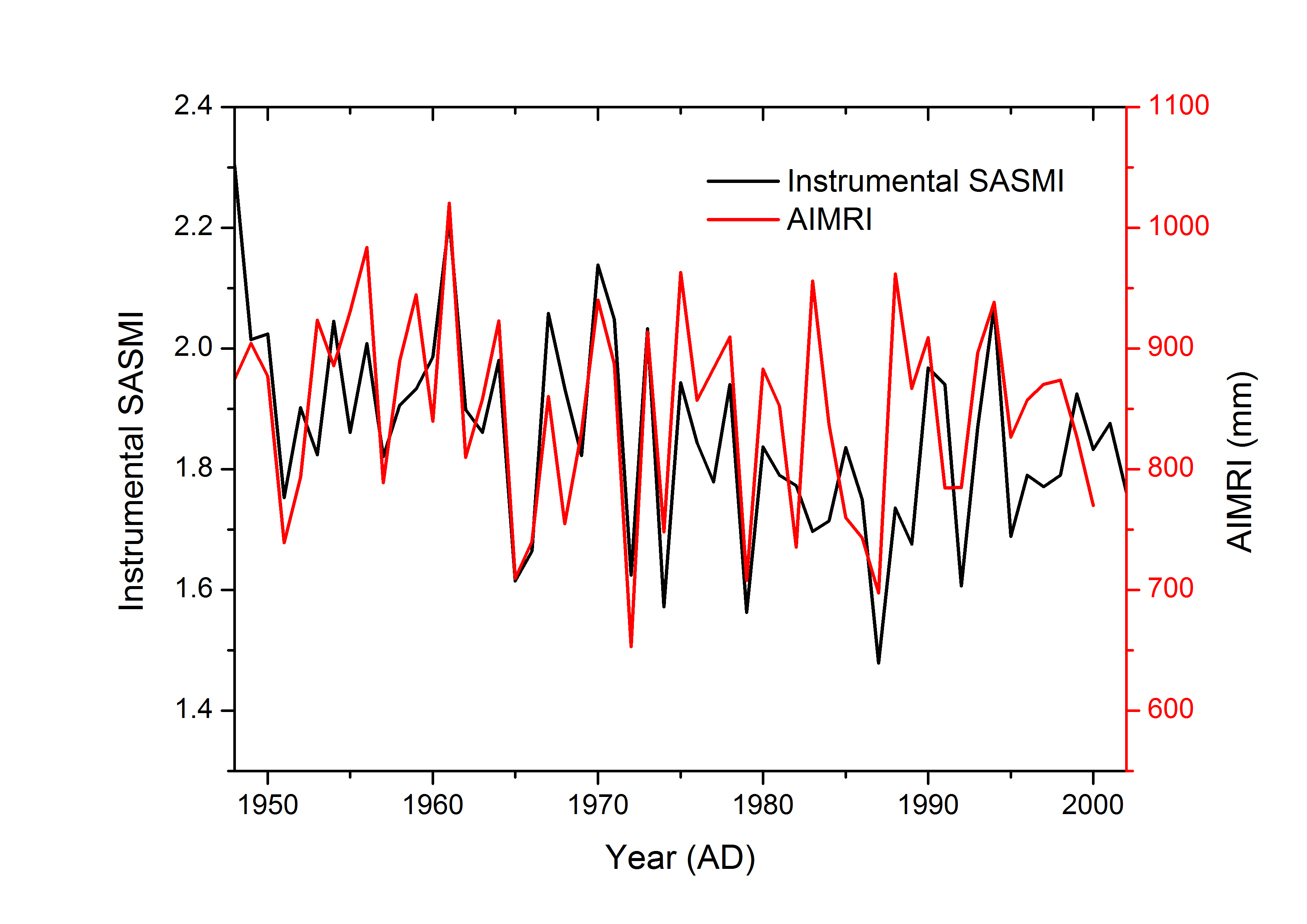


Figure S3. Time series of the all-India monsoon rainfall index (AIMRI)(red line) and the instrumental South Asian summer monsoon index (SASMI) (black line).

Figure S4. Correlations between the South Asian summer monsoon index (SASMI) and four common instrumental precipitation datasets, all of which are significant at the 90% confidence level. The green and white points represent the screened tree-ring chronologies that are positively and negatively correlated with the SASMI at the 90% confidence level. The numbers of grids showing positive or negative correlations is noted. Figure S4 was generated using the NCAR Command Language (Version 6.1.2) [Software] ((2013). Boulder, Colorado: UCAR/NCAR/CISL/VETS. http://dx.doi.org/10.5065/D6WD3XH5).

References:

1. Thompson L*, et al.* A high-resolution millennial record of the South Asian monsoon from Himalayan ice cores. *Science***289**, 1916-1919 (2000).

2. Sinha A*, et al.* The leading mode of Indian Summer Monsoon precipitation variability during the last millennium. *Geophys. Res. Lett.* **38***,* doi:10.1029/2011gl047713 (2011).

3. Buckley B*, et al.* Climate as a contributing factor in the demise of Angkor, Cambodia. *P Natl Acad Sci USA***107**, 6748-6752 (2010).

4. Buckley B, Palakit K, Duangsathaporn K, Sanguantham P, Prasomsin P. Decadal scale droughts over northwestern Thailand over the past 448 years: links to the tropical Pacific and Indian Ocean sectors. *Clim Dyn***29**, 63-71 (2007).

5. Miller FP, Vandome AF, John MB. [Famine in India] (VDM Publishing, 2010).

6. Loveday A. [The history and economics of Indian famines] (G. Bell and sons LTD., London, 1914).

7. Murton B. [Spatial and Temporal Patterns of Famine in Southern India before the Famine Codes] *Famine as a geographical phenomenon* [Currey B, Hugo, G (ed.)] (D. Reidel Publishing Company, Dordrecht, 1984).

8. Currey B, Hugo G. [Famine: As a geographical phenomenon] *The GeoJournal Library* (Springer, 1984).

9. Sen A. Public action to remedy hunger. *Interdiscipl Sci Rev***16**, 324-336 (1991).

10. Gráda C. Making famine history. *J. Econ Lit*, **45**,5-38(2007).

11. Rathore NS. A Historical Perspective of the Development of Rain Water Harvesting Techniques in the Mewar Region, Udaipur, Rajasthan, India. *Int J. Water Resour Arid Environer,***1**, 285-294(2011).

12. Bhatia BM. [Famines in India. A study in some aspects of the economic history of India (1860-1965)](Asia Publ. House, London 1967).

13. Fukazawa H, Fukazawa H. [Maharashtra and the DeccanThe Cambridge Economic History of India] (Cambridge University Press, 1982).

14. Nash M. El Niño: unlocking the secrets of the master weather-maker. Conference: Fermilab Colloquia, Fermi National Accelerator Laboratory (FNAL), Batvia, Illinois (United States) (2002).

15. Collier M, Webb RH. [Floods, droughts, and climate change] (University of Arizona Press, 2002).

16. Leiberman V. [Strange Parallels: vol. 1, Integration on the Mainland:Southeast Asia in Global Context, C. 800–1830] (Cambridge University Press, 2003).

17. Grove RH. The Great El Niño of 1789–93 and its Global Consequences Reconstructing an Extreme Climate Event in World Environmental History. *Med Hist J.***10**, 75-98 (2007).

18. Balfour E. [The Cyclopædia of India and of Eastern and Southern Asia: Commercial, Industrial and Scientific, Products of the Mineral, Vegetable, and Animal Kingdoms, Useful Arts and Manufactures] (B. Quartitch, 1885).

19. Derbyshire I. Economic Change and the Railways in North India, 1860-1914. *Mod Asian Stud***21**, 521-545 (1987).

20. Butler SH. [Famine]*The Imperial Gazetteer of India, the Indian empire, vol. III Economic*[Henry F. (eds)] [473-506](The Clarendon Press, 1908).

21. Habib I.[Population]*The Cambridge economic history of India c.1200-c.1750* [Raychaudhuri T. & Habib I. (ed.)] [161-171] (Cambridge University Press, 1982).

22. Davis M. [Late Victorian holocausts: El Niño famines and the making of the third world] (Verso, London, 2001).

23. Sami L. Gender Differentials in Famine Mortality: Madras (1876-78) and Punjab (1896-97). *Econ Polit Weekly***37**, 2593-2600 (2002).

24. BREWIS G. ‘Fill Full the Mouth of Famine’: Voluntary Action in Famine Relief in India 1896–1901. *Mod Asian Stud***44**, 887-918 (2010).

25. Dyson T, Maharatna A. Bihar Famine, 1966-67 and Maharashtra Drought, 1970-73: The Demographic Consequences. *Econ Polit Weekly***27**, 1325-1332 (1992).

26. Parthasarathy B, Sontakke N, Monot A, Kothawale D. Droughts/floods in the summer monsoon season over different meteorological subdivisions of India for the period 1871–1984. *J. Climatol***7**, 57-70 (1987).

27. Kumar RH*, et al.* Diet and Nutritional Situation of the Population in the Severely Drought Affected Areas of Gujarat. *J. Hum Ecol***18**, 319-326 (2005).

28. PAGES 2k Consortium. Continental-scale temperature variability during the past two millennia. *Nat Geosci***6**, 339-346 (2013).

29. Sano M, Buckley B, Sweda T. Tree-ring based hydroclimate reconstruction over northern Vietnam from Fokienia hodginsii: eighteenth century mega-drought and tropical Pacific influence. *Clim Dyn***33**, 331-340 (2009).

30. Li J, Yuan Y, You X. [The Tree-ring Hydrology Research and Application] (Science Press, Beijing, 2000).

31. Yang B, Qin C, Shi F, Sonechkin DM. Tree ring-based annual streamflow reconstruction for the Heihe River in arid northwestern China from AD 575 and its implications for water resource management. *Holocene***22**, 773-784 (2012).

32. Xu C, Sano M, Nakatsuka T. Tree ring cellulose δ18O of Fokienia hodginsii in northern Laos: A promising proxy to reconstruct ENSO? *J. Geophys. Res.* **116***,* doi:10.1029/2011JD016694 (2011).

33. Xu C, Sano M, Nakatsuka T. A 400-year record of hydroclimate variability and local ENSO history in northern Southeast Asia inferred from tree-ring δ18O. *Palaeogeogr Palaeocl***386**, 588-598 (2013).
